# Whole-body MRI of patients with polymyalgia rheumatica identifies a distinct subset with complete patient-reported response to glucocorticoids

**DOI:** 10.1136/annrheumdis-2015-207395

**Published:** 2015-09-16

**Authors:** Sarah Louise Mackie, Colin Thomas Pease, Eiji Fukuba, Emma Harris, Paul Emery, Richard Hodgson, Jane Freeston, Dennis McGonagle

**Affiliations:** 1 Leeds Institute for Rheumatic and Musculoskeletal Medicine, Leeds, UK; 2 NIHR-Leeds Musculoskeletal Biomedical Research Unit, Leeds, UK; 3 Leeds Teaching Hospitals NHS Trust, Leeds, UK; 4 Department of Radiology, Shimane University, Izumo, Japan; 5 University of Manchester Centre for Imaging Sciences, Manchester, UK

**Keywords:** Magnetic Resonance Imaging, Polymyalgia Rheumatica, Cytokines

## Abstract

**Objectives:**

To determine whether whole-body MRI defines clinically relevant subgroups within polymyalgia rheumatica (PMR) including glucocorticoid responsiveness.

**Methods:**

22 patients with PMR and 16 with rheumatoid arthritis (RA), untreated and diagnosed by consultant rheumatologists, underwent whole-body, multiple-joint MRI, scored by two experts. Patients with PMR reported whether they felt ‘back to normal’ on glucocorticoid therapy and were followed for a median of 2 years.

**Results:**

All patients with PMR were deemed to respond to glucocorticoids clinically. A characteristic pattern of symmetrical, extracapsular inflammation, adjacent to greater trochanter, acetabulum, ischial tuberosity and/or symphysis pubis, was observed in 14/22 of the PMR cases. In PMR, this pattern was associated with complete glucocorticoid response (p=0.01), higher pretreatment C-reactive protein (CRP) and serum interleukin-6 (IL-6), and better post-treatment fatigue and function. Only 1/14 in the extracapsular group could stop glucocorticoids within 1 year, compared with 4/7 of the others. A score derived from the five sites discriminating best between PMR and RA correlated with IL-6 (p<0.002). IL-6 levels ≥16.8 pg/mL had 86% sensitivity and 86% specificity for the extracapsular MRI pattern.

**Conclusions:**

A subset of patients with rheumatologist-diagnosed PMR had a characteristic, extracapsular pattern of MRI inflammation, associated with elevated IL-6/CRP and with complete patient-reported glucocorticoid responsiveness.

## Introduction

Polymyalgia rheumatica (PMR) is a clinically diagnosed cause of glucocorticoid-responsive pain and stiffness at the shoulders and hips, with great variation in the duration of glucocorticoid treatment required.^1^ ^2^ Previous MRI and 18-fluorodeoxyglucose (FDG)-positron emission tomography (PET) studies have suggested distinct extracapsular^3^ ^4^ or capsular-based^5^ inflammation in PMR. Elevated pretreatment interleukin-6 (IL-6) levels (>10 pg/mL) with good symptomatic response to 20 mg prednisone was associated with requirement for >1 year of therapy.^6^ Given the superior resolution of MRI compared with 18-FDG-PET, we sought to determine an anatomical explanation for these findings.

Rheumatologists have traditionally been concerned not to miss rheumatoid arthritis (RA) in patients with PMR, although the anti-citrullinated peptide (anti-CCP) antibody test has made this easier.^7^ ^8^ We designed this study to identify patterns of inflammation on whole-body, multiple-joint, 3-Tesla MRI^9^ that distinguished PMR from RA but during follow-up we were struck by the prognostic heterogeneity within the PMR group. Given a known association of ultrasound-defined inflammation with glucocorticoid responsiveness in PMR,^10^ we hypothesised that an extracapsular pattern of inflammation in PMR predicts glucocorticoid response.

## Methods

Ethical approval was obtained (09/H1307/98, approved by Leeds West Research Ethics Committee 15.1.10; 05/Q1108.28, York Research Ethics Committee). All patients gave written, informed consent.

### Cases

Twenty-two consecutive patients with untreated PMR fulfilling Bird criteria^11^ were identified by two rheumatologists. All had an elevation of at least one acute-phase marker (C-reactive protein (CRP), erythrocyte sedimentation rate (ESR) plasma viscosity (PV)), were negative for rheumatoid factor and anti-CCP antibody and were commenced on prednisolone 15 mg after their MRI scan, increasing to 20 mg at 1-month follow-up if clinically indicated.

Patients recorded pain/stiffness location using mannequins, and graded symptom severity using visual analogue scores (VAS) and Stanford Health Assessment Questionnaire - Disability Index (HAQ-DI).^12^ Patients were asked whether they felt ‘back to normal since taking steroids’, on a five-point Likert scale from ‘strongly agree’ to ‘strongly disagree’. ‘Strongly agree’ and ‘agree’ were classified as ‘yes’, others ‘no’. Standardised glucocorticoid taper was adjusted to maintain symptom control until glucocorticoid cessation.^2^ Median follow-up was 2 years.

### Imaging controls

To minimise MRI scorer bias, 16 control MRI scans were chosen from patients with seropositive or seronegative RA.

### MRI

**Figure 1:**
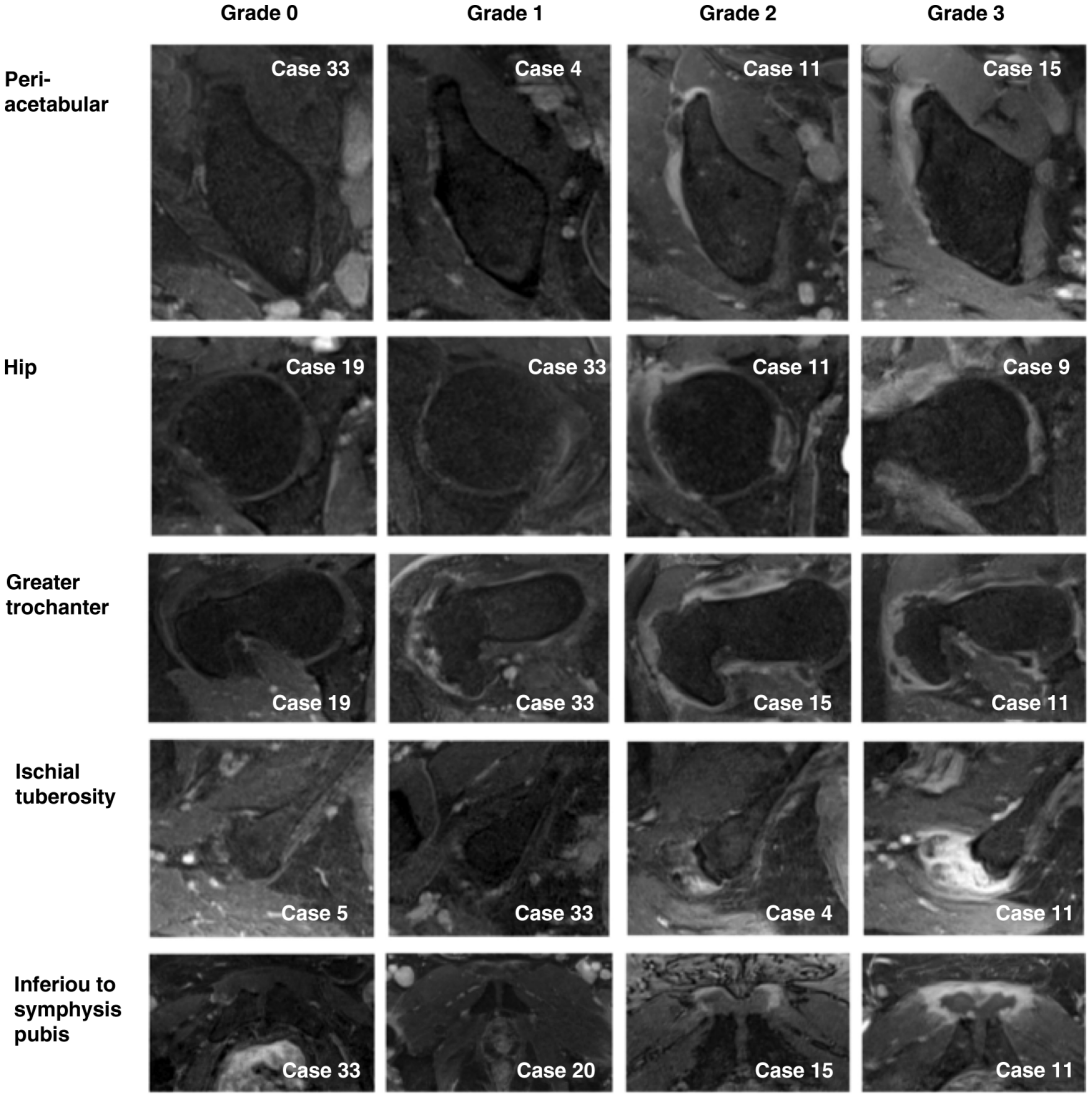
Exemplar images of semiquantitative scoring system.

Whole-body multiple-joint MRI was performed^9^ (see online supplementary methods). Gadolinium was used except where contraindicated. The four non-contrast MRI image files were evaluated (by DM, SLM and EF) to determine presence/absence of extracapsular PMR pattern as previously described.^3^ All 34 whole-body, multiple-joint, contrast-enhanced MRI image files were anonymised. Axial images were systematically scored in ImageJ in the following order: spine, shoulders, hips, hands, knees, feet. Each defined site was semiquantitatively consensus scored by the two experts (DM and EF), scoring 0, 1, 2 or 3, for no inflammation, mild, moderate or severe inflammation respectively (figure 1). Each MRI was also classified as ‘extracapsular pattern’ or ‘non-extracapsular pattern’. The anonymisation code was not broken until the MRI scoring datasheet (including overall classification) had been locked down.

### IL-6 measurement

IL-6 was measured by ELISA (IL-6 Quantikine, R+D Systems) using serum taken from consenting patients before MRI.

### Analysis

We tested the hypothesis that an extracapsular pattern of disease was associated with glucocorticoid responsiveness. Statistical analyses were performed in SPSS V.21 (IBM).

## Results

### Demographics and disease characteristics

At screening, all 22 patients with PMR fulfilled Bird criteria^11^ and (retrospectively) the provisional ACR/EULAR classification criteria,^8^ including elevation of at least one acute-phase marker; in two PMR cases, however, acute-phase markers normalised by the time the MRI scan was done. At follow-up, PMR was confirmed as the most likely diagnosis. All 22 patients were recorded by the treating rheumatologist as responding to prednisolone; in three cases an increase in dose was required for complete response. No alternative explanation for patients’ musculoskeletal symptoms was found.

### Training set (non-contrast) MRIs in PMR

Extracapsular inflammation^3^ was seen in 2/4 non-contrast MRI scans of patients with PMR. Oedema was seen around the greater trochanter in both, and of subdeltoid bursa, glenohumeral joint and below the symphysis pubis in one. There was no difference between the patients with PMR who did and did not receive contrast (see online supplementary table S2).

### Gadolinium-enhanced whole-body multiple-joint MRI

**Figure 2:**
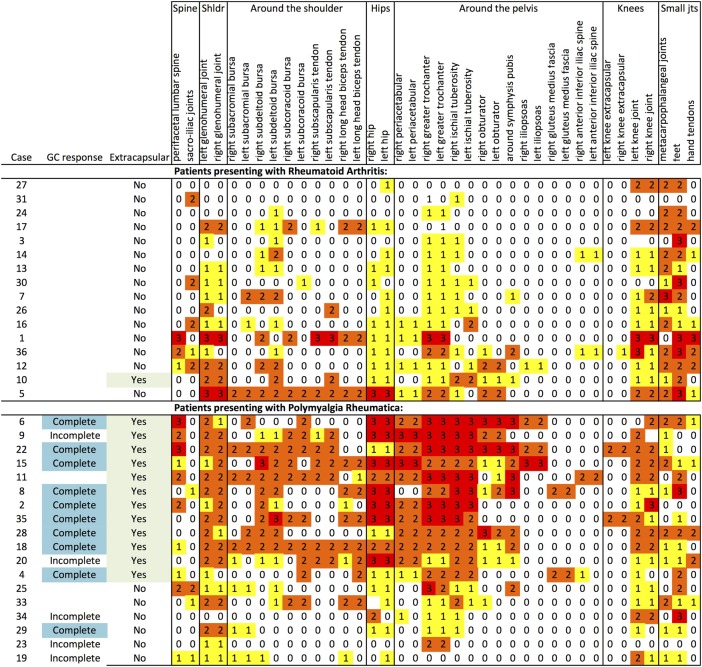
Results of blinded scoring of gadolinium-enhanced MRI scans. Prosthetic joints are treated as missing data (cells left blank). GC, glucocorticoid.

The same extracapsular pattern was seen on contrast MRI as with non-contrast MRI. Inflammation around the shoulders was seen in both PMR and RA; PMR additionally featured pelvic inflammation especially adjacent to greater trochanter and ischial tuberosity (see online supplementary figure S1) and periacetabular anterolateral to the rim of the acetabulum, without involving the synovial hip joint and extending superiorly from the anterior hip capsule, medial to gluteus minimus and lateral to the iliac bone, not typical for iliopectinal (iliopsoas) bursitis (see online supplementary figure S1). We additionally observed inflammation inferior to the symphysis pubis (see online supplementary figure S1). Bone oedema was absent. 14/22 of the patients with PMR and 1/16 of the patients with RA were classified as ‘extracapsular pattern’ (p<0.001). Results of semiquantitative scoring (figure 1) are shown in figure 2.

### Clinical associations of extracapsular pattern

Prior to glucocorticoid therapy, according to the rheumatologists’ assessments, the extracapsular group and the non-extracapsular group appeared to have the same clinical syndrome of PMR with no difference in the PMR-AS activity score.^13^ The non-extracapsular group was all women, and had a lower CRP and IL-6 (table 1). Males had a higher MRI inflammation score (p=0.046) and a lower stiffness VAS (p=0.037), non-significant after Bonferroni correction (data not shown). 9/13 of those scoring 2 or 3 at ischial tuberosity indicated buttock pain on pain/stiffness mannequins, compared with 0/5 of the remainder (sensitivity 69%, specificity 100%) (see online supplementary figure S3).

**Table 1:** Description of features of patients with polymyalgia rheumatica (PMR) with and without characteristic extracapsular pattern of inflammation

	Extracapsular pattern (n=14)	Non-extracapsular pattern (n=8)	p Value
Demographics			
Age, median (range)	75 (55, 85)	78 (70, 84)	0.22
Male, n (%)	8 (57%)	0 (0%)	0.02
Acute-phase markers			
ESR, median (range), mm/h	46 (9, 119)	38 (4, 81)	0.63
CRP, median (range), mg/L	36 (5, 118)	5.25 (5, 76)	0.03
PV, median (range), mPa s	1.93 (1.78, 2.12)	1.81 (1.57, 2.04)	0.36
IL-6, median (range), pg/mL	25.8 (0.3, 87.6)	6.0 (0.2, 131.5)*	0.04
Composite disease activity scores			
PMR-AS (median, IQR)	78.6 (53.8, 103.0)	70.2 (34.4, 106.1)	0.73
Pretreatment patient-reported outcomes			
Pain VAS (median, IQR)	7.7 (5.0, 8.1)	7.9 (4.3, 8.7)	0.63
Stiffness VAS (median, IQR)	6.3 (4.5, 8.0)	8.2 (5.2, 8.9)	0.29
Fatigue VAS (median, IQR)	7.1 (5.2, 7.6)	8.4 (7.0, 9.7)	0.03
HAQ-DI (median, IQR)	1.25 (1.09, 1.50)	1.56 (1.28, 2.09)	0.07
Assessment of glucocorticoid responsiveness at first follow-up			
‘I feel back to normal since taking steroids’. n (%)	11/13 (85%)	1/6 (17%)	0.01
‘I feel [or felt] back to normal since taking steroids’. n (%)	12/14 (86%)	1/8 (13%)	0.001
Fatigue VAS at follow-up (median, IQR)	1.3 (0.2, 3.8)	7.1 (3.6, 9.8)	0.02
HAQ-DI at follow-up (median, IQR)	0 (0, 0.625)	1.0 (0.76, 2.07)	0.003
Prognosis			
Stopped glucocorticoids permanently after <1 year†	1/14	4/7	0.03
Relapse-free†	7/14	2/8	1.00
Relapsed when on 5 mg or more†	3/14	3/7	0.35
Required initial dose increase >15 mg	2/14	1/8	1.00

The PMR-AS is the PMR Activity Score as described by Bird and Leeb. Either Mann–Whitney U test or Fisher's exact test was used for non-normally distributed values; unpaired t test for normally distributed variables. All tests were two-tailed. Apart from glucocorticoid responsiveness (the a priori hypothesis), p values should be interpreted in the light of multiple testing. Bonferroni correction for all the variables reported here (likely over-stringent because of strong correlation between ESR/CRP/PV/IL-6 and between patient-reported VAS scores) would require a threshold of 0.05/19, or p<0.0026.

*Excludes one patient who did not have IL-6 measured.

†Excludes one patient who was lost to follow-up at 4 months.

CRP, C-reactive protein; IL-6, interleukin-6; PV, plasma viscosity; VAS, visual analogue score.

Where questionnaire data were available from first follow-up (not done in three because of time constraints), 11/13 of those with characteristic ‘extracapsular pattern’ of MRI inflammation, and 1/6 of the remainder, stated that they felt ‘back to normal’ since taking steroids (p=0.01). This was reflected in better patient-reported function (HAQ-DI) and fatigue VAS (table 1) but no difference in post-treatment pain or stiffness VAS between patients with and without the extracapsular pattern (data not shown). The remaining three patients were later asked whether they had felt ‘back to normal’ in the first month after taking steroids; therefore, 12/14 of all the patients with PMR an extracapsular pattern felt ‘back to normal’, compared with 1/8 of those without an extracapsular pattern (p=0.001). Those with an extracapsular pattern were less likely to be able to stop glucocorticoid therapy within the first year.

The two patients with PMR extracapsular pattern who were not complete glucocorticoid responders by self-report (figure 2) had the highest IL-6 and CRP, and both required escalation of prednisolone dose for full response. Another patient with extracapsular pattern later developed biopsy-proven giant cell arteritis.

### Association of IL-6 with MRI inflammation

The top five MRI features (mean of left and right) were summed to provide a composite score. This was significantly associated with IL-6 (p<0.001) (see online supplementary figure S2) but not with CRP (p=0.055). The most discriminatory IL-6 cut-off for the extracapsular pattern was ≥16.8 pg/mL (sensitivity 86%, specificity 86%).

## Discussion

All our patients were diagnosed with PMR by rheumatologists; we sought to determine whether this could be further stratified based on the pattern and extent of inflammation on whole-body MRI. We identified a subset, with characteristic, extracapsular pattern of inflammation on MRI that was more likely to feel ‘back to normal’ after glucocorticoids. MRI allowed good resolution of pelvic inflammation. In addition, despite having more males (male gender in PMR generally predicts shorter glucocorticoid duration^14^), our ‘extracapsular’ group was also more likely to require glucocorticoid treatment for >1 year. IL-6 correlated with pelvic MRI inflammation; a cut-off of ≥16.8 pg/mL IL-6 had 86% sensitivity and 86% specificity for the extracapsular pattern. Our data support an extra-articular model of the primary inflammatory change in PMR.^3^ A recent report describes focal 18-FDG-PET uptake anterior to the hip joint in PMR^15^ similar to our ‘periacetabular’ pattern.

Strengths of this study include the standardised assessments and the blinded MRI scoring. PMR diagnoses were all made by a consultant rheumatologist, and all patients were treated as PMR without any alternative diagnosis supervening.

The limitations of this study were its descriptive and exploratory nature, small numbers, slightly younger age of the RA group, and the subjectivity inherent in clinical diagnosis of PMR even following diagnostic guidelines.^8^ ^16^ We hypothesise the ‘non-extracapsular’ patients with PMR may be a pathogenetically heterogeneous group, analogous to ‘autoantibody-negative RA’. Whether they ought to be labelled PMR is a philosophical question beyond the scope of this investigation.

This novel, pathoanatomical description of the clinical spectrum of PMR adds weight to the idea of PMR as a clinically heterogeneous disorder.^1^ MRI may help to identify a more homogeneous subset, with potential value for defining eligibility for early clinical trials of targeted therapies. Our data suggest that in the specialist setting CRP and IL-6 may be more prognostically useful tests than ESR or PV. An MRI might be useful in cases of diagnostic doubt. The diagnostic importance of glucocorticoid responsiveness is still debated in PMR^8^ ^17^ especially since the best way to measure response remains unclear: the limitations of previously proposed disease activity scores, including the physician global assessment and the PMR-AS, have been well-discussed elsewhere.^10^ Although most disease activity scores focus on pain and stiffness, we identified residual fatigue and functional impairment after glucocorticoid treatment in our non-extracapsular group; this is of interest since fatigue^18^ and disability^19^ have been identified from recent qualitative studies as being important to patients. We found that MRI yielded additional valuable information to the clinical assessment, particularly in the pelvic region where an extracapsular pattern was clearly seen in a distinct subset of patients. Further research is required to determine the clinical utility of MRI, symptom location (eg, buttock pain) or CRP/IL-6 as diagnostic, prognostic or treatment stratification features in PMR.

## Supplementary Material

Web supplement
