# Supplementary material for: Whole-body MRI of patients with polymyalgia rheumatica identifies a distinct subset with complete patient-reported response to glucocorticoids
Source: Ann Rheum Dis. 2015 Sep 16;74(12):2188–92. doi: 10.1136/annrheumdis-2015-207395 (PMC4680120; doi:10.1136/annrheumdis-2015-207395)

Online-Only Supplement – Contents and Legends

Supplementary Methods. Sequence parameters for 3-Tesla, whole-body, multiple-joint magnetic resonance imaging scans.

Supplementary Table 1. Clinical and demographic features of cases and controls at recruitment.

Supplementary Table 2. Discrimination between PMR and RA of individual scores at 13 regions in the shoulder or hip.

Supplementary Figure 1. Trios of images from a single case (no. 11) illustrating some of the characteristic features of the extracapsular inflammation pattern. Coloured lines represent the three intersecting orthogonal planes (axial, sagittal or coronal) used to generate each trio of images. (a) Cross sections through femoral head showing peri-acetabular enhancement (b) Cross sections through pubic bone showing enhancement below the symphysis pubis; also showing inflammation of anterior superior iliac spine.

Supplementary Figure 2. Relationship of serum IL-6 to MRI score. Spearman rho=0.754, p<0.001, n=17.

Supplementary Figure 3. Pain (red) overlaid with stiffness (green) mannequins, for each of the patients who underwent contrast-enhanced MRI scans.

Supplementary Methods.

Sequence parameters for 3-Tesla, whole-body, multiple-joint magnetic resonance imaging scans.

	TR/TE/FA	Resolution (mm)	Time (mins)	
Spine x3	3100/105	1.2 x 0.9 x 3	1:15	2D FSE SPAIR
Sacro-iliac joints	4580/107	1.1 x 0.9 x 3	1:33	
Shoulders	11/2.5, 3.7/15	1.0 x 1.0 x 0.6	3:02	3D VIBE Dixon post gadolinium
Hips, hands	6.4/2.5, 3.7/15	0.8 x 0.8 x 0.6	3:02	
Knees	10/2.4, 3.7/20	0.8 x 0.8 x 0.6	2:59	
Feet	6.4/2.5, 3.7/15	0.8 x 0.8 x 0.6	3:16	

Whole-body multiple-joint MRI was performed using a 3T Siemens Verio scanner with multiple radiofrequency coils. T2-weighted, fat-suppressed images of the spine and sacroiliac joints were acquired, followed by images of the joints and entheses (regions: shoulders, hips and hands, knees, feet) using VIBE (volumetric interpolated breath hold examination) Dixon sequences post intravenous gadolinium contrast. If gadolinium was contra-indicated, a non-contrast scan covering equivalent anatomical areas was performed.

Supplementary Table 1. Clinical and demographic features of cases and controls at recruitment.

	PMR cases having MRI	PMR cases having contrast MRI	RA controls having contrast MRI
Total number	22	18	16
Males (n, %)	8 (36.4%)	7 (38.9%)	6 (37.5%)
Age (median, range)	75.5 (55, 85)	75.5 (55, 85)	52.5 (23, 66)
C-reactive protein, mg/L (median, interquartile range)	30 (5.4, 55)	30 (<5.0, 43.8)	13 (<5.0, 21.1)
ESR, mm/hour (median, interquartile range)	46 (22.25, 54.5)	42 (17, 54.5)	23.5 (6, 37.5)
Plasma viscosity, mPa s (median, interquartile range)*	1.84 (1.79, 1.98)	1.84 (1.79, 1.98)	1.82 (1.69, 1.89)
IL-6, pg/mL (median, interquartile range)	21.1 (5.2, 31.4)	21.1 (2.9, 31.0)	-
Seropositive***, n (%)	0	0	10 (62.5%)
Radiographic erosions present, n (%)	0	0	8 (50%)
Pain VAS, (median, interquartile range)	7.7 (5.0, 8.5)	7.7 (5.0, 8.5)	-
Stiffness VAS, (median, interquartile range)	6.9 (4.9, 8.75)	6.9 (4.9, 8.75)	-
Fatigue VAS, (median, interquartile range)	7.3 (6.1, 8.3)	7.3 (5.7, 8.5)	-
HAQ-DI, (median, interquartile range)	1.375 (1.125, 1.719)	1.312 (1.125, 2.00)	-

*Reference range for plasma viscosity in our laboratory: 1.50 to 1.72.
**Excludes one PMR patient who did not have IL-6 measured.

***Seropositivity defined as rheumatoid factor titres and/or anti-cyclic citrullinated peptide antibody levels outside the quoted laboratory reference range.

Supplementary Table 2. Discrimination between PMR and RA of individual scores at 13 regions in the shoulder or hip.

Site	Region	c-statistic	95 % CI of c-statistic
Ischial tuberosity	Pelvic	0.833	0.689, 0.977
Hip joint	Pelvic	0.785	0.628, 0.941
Greater trochanter	Pelvic	0.780	0.617, 0.942
Peri-acetabular	Pelvic	0.771	0.609, 0.933
Symphysis pubis	Pelvic	0.689	0.510, 0.869
Subcoracoid bursitis	Shoulder	0.684	0.503, 0.865
Subacromial bursitis	Shoulder	0.663	0.478, 0.848
Obturator tendon	Pelvic	0.661	0.477, 0.846
Long head of biceps tendon	Shoulder	0.658	0.469, 0.847
Glenohumeral joint synovitis	Shoulder	0.656	0.457, 0.855
Subdeltoid bursitis	Shoulder	0.573	0.378, 0.768
Iliopsoas bursitis	Pelvic	0.557	0.362, 0.752
Subscapularis tendon	Shoulder	0.530	0.727, 0.332

For bilateral structures the mean of the scores from the right and left are taken. The value of the c-statistic, equivalent to the area under the ROC curve, is defined as 0.500 for a non-discriminatory test (no better than chance) and 1.000 for a perfectly discriminatory test. Sites of inflammation are ranked in descending order of the c-statistic.

Supplementary Figure 1a,b. Trios of images from a single case (no. 11) illustrating some of the characteristic features of the extracapsular inflammation pattern. Coloured lines represent the three intersecting orthogonal planes (axial, sagittal or coronal) used to generate each trio of images.

(a) Cross sections through femoral head showing peri-acetabular enhancement

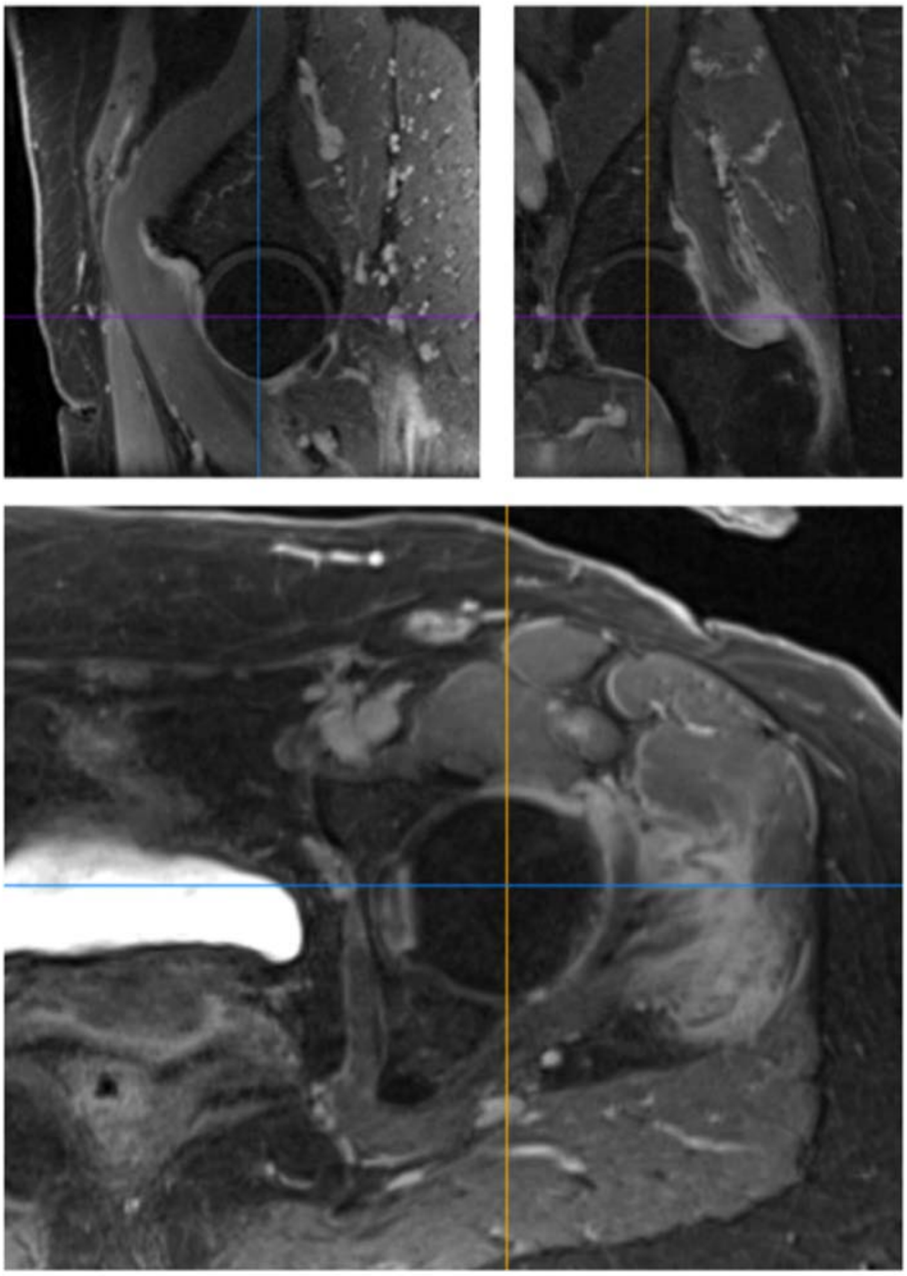

(b) Cross sections through pubic bone showing enhancement below the symphysis pubis; also showing inflammation of anterior superior iliac spine.

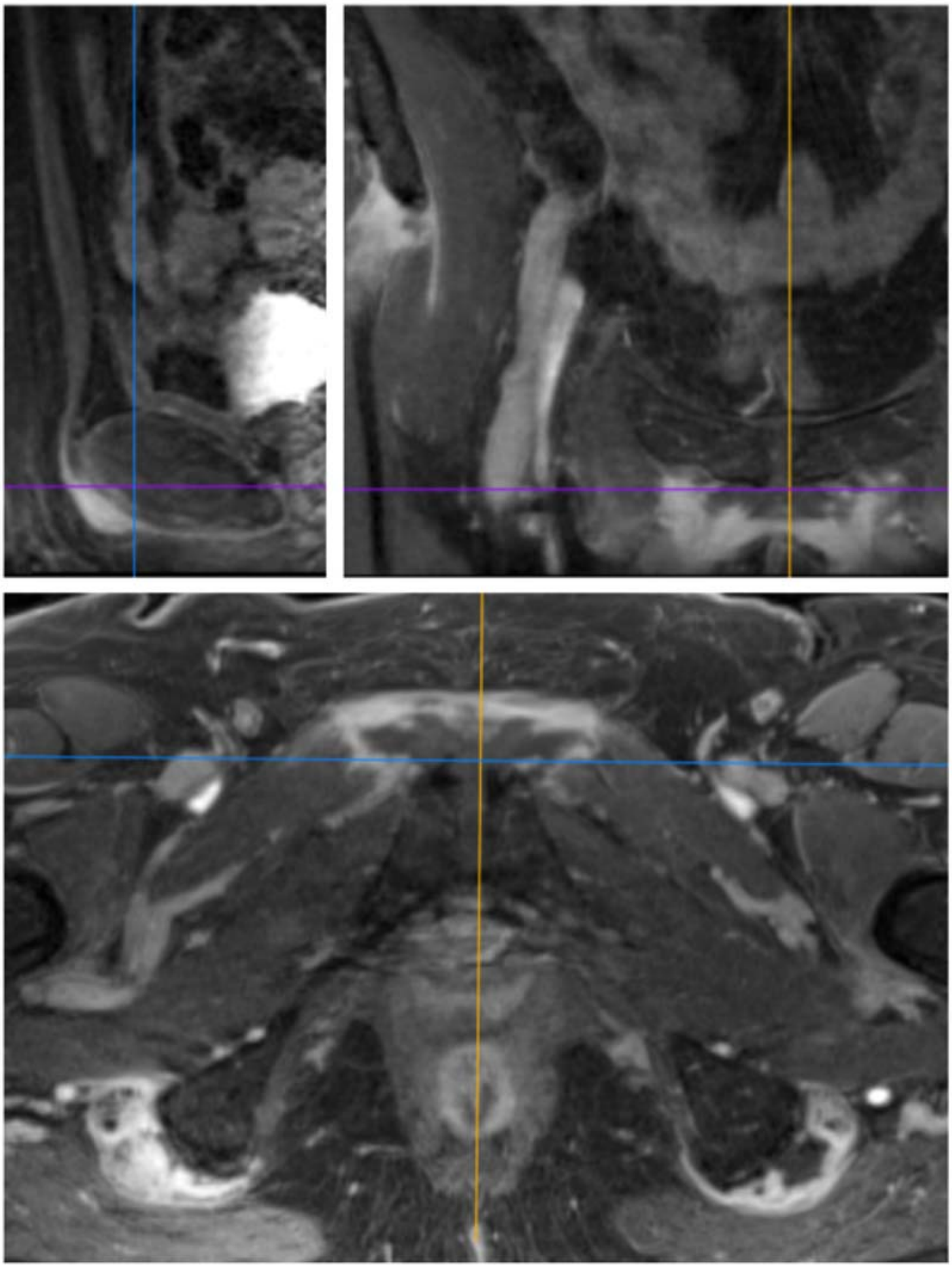

Supplementary Figure 2. Relationship of serum IL-6 to MRI score. Spearman rho=0.754, p<0.001, n=17

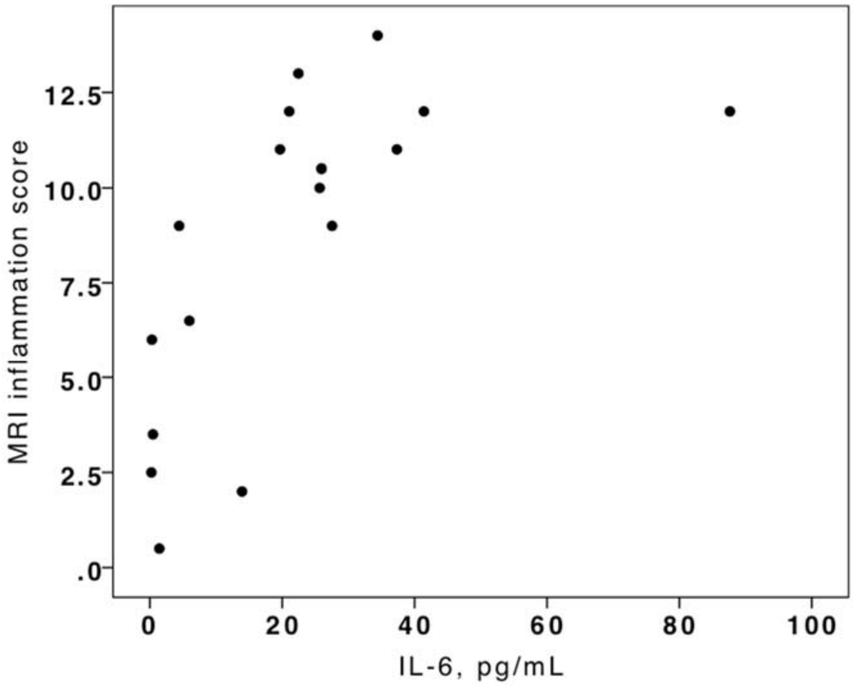

Supplementary Figure 3. Pain (red) overlaid with stiffness (green) mannequins, for each of the patients who underwent contrast-enhanced MRI scans.

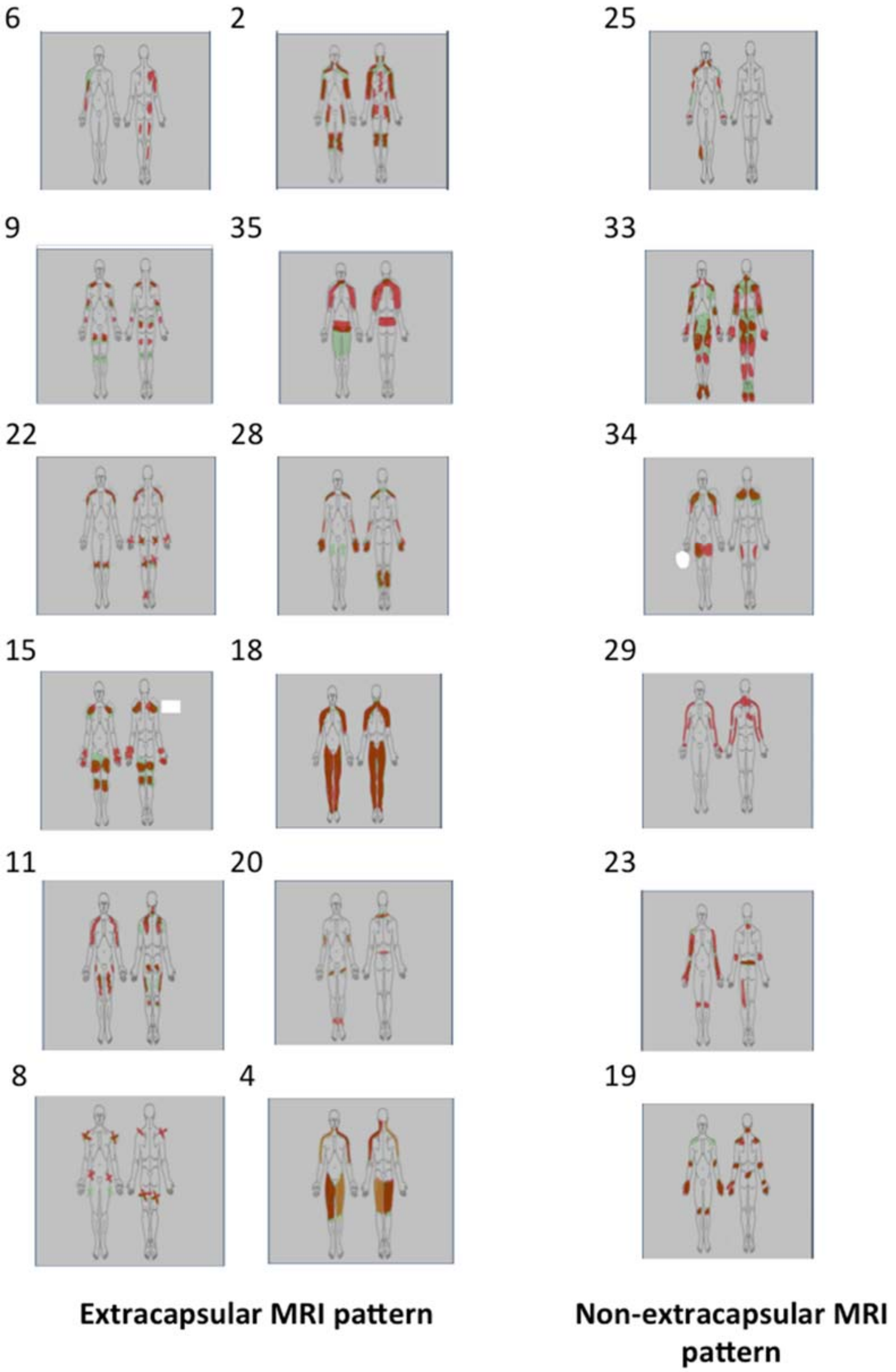

Supplement: Web supplement [file annrheumdis-2015-207395-s1.pdf]
